# Sleep Health Promotion in the Workplace

**DOI:** 10.3390/ijerph17217952

**Published:** 2020-10-29

**Authors:** Sergio Garbarino, Giovanni Tripepi, Nicola Magnavita

**Affiliations:** 1 Post-Graduate School of Occupational Medicine, Università Cattolica del Sacro Cuore, 00168 Rome, Italy; nicola.magnavita@unicatt.it or; 2 State Police Health Service Department, Ministry of the Interior, 00198 Rome, Italy; 3 Department of Neuroscience, Rehabilitation, Ophthalmology, Genetics and Maternal-Infantile Sciences, University of Genoa, 16126 Genoa, Italy; 4 Research Unit of Reggio Calabria, Institute of Clinical Physiology, Italian National Research Council, 89124 Calabria, Italy; gtripepi@ifc.cnr.it; 5 Department of Woman/Child & Public Health, Fondazione Policlinico Agostino Gemelli IRCCS, 00168 Rome, Italy

**Keywords:** workplace health promotion, sleep quality, sleep hygiene, sleepiness, safety, insomnia, sleep deprivation, injury, near-miss, police

## Abstract

Poor sleep and sleepiness in the workplace are associated with accidents. A workplace sleep health promotion program was implemented in an Italian police unit. Of the 242 police officers in the unit, 218 (90%) agreed to take part in the program. A crossover trial was made in which the police officers were divided into two groups that performed sleep health promotion activities in the first and second year, respectively. The first group of officers showed significant sleep improvements at the end of the first year, while the second group had similar or worse parameters than at baseline. At follow-up, a significant improvement in the quantity and quality of sleep was reported in both groups. Sleep improvements at follow-up were associated with a marked reduction in the frequency of accidents at work and near-misses. Before the intervention, sleepiness was the best predictor of injuries (aOR 1.220; CI95% 1.044–1.426) and near-misses (aOR 1.382; CI95% 1.182–1.615). At follow-up, when sleep conditions had improved, insomnia symptoms were the most significant predictors of work accidents (aOR 13.358; CI95% 2.353–75.818). Sleep health promotion can be useful in police officers.

## 1. Introduction

An abnormal quantity or quality of sleep is associated with many diseases, such as cardiovascular disorders, diabetes, psychiatric, and behavioral disorders, and all-cause mortality [1], and a reduced wellbeing [2]. There is also evidence that poor sleepers are more frequently absent from work, have more accidents, and are less productive [3]. The possible damage caused by a reduction in the quantity or quality of sleep can be more serious in the caring professions since not only does it harm the worker, but it can also affect the people who benefit from their assistance. An example of this can be observed in police work.

The importance of sleep in the police force has led many research groups to focus on this topic. The literature indicates that police officers often have sleep problems. A systematic meta-analysis review indicated that the pooled prevalence of bad sleep quality in police officers was 51% (95%CI 42–60%) [4]. Several researchers have therefore proposed programs to improve the health of police officers [5]. A worksite wellness program among police officers proved to be effective in improving diet, sleep, stress, and the overall quality of life [6]. Similarly, a fatigue management training program [7] and psychological support [8] resulted in positive sleep health benefits for police workers. A systematic review of health promotion studies showed that structured programs together with training, support during behavior change, and peer support had the greatest impact on sleep quality [5]. Although health promotion intervention encounters numerous barriers, this type of action is increasingly important on account of the aging of the workforce that also affects the police [9]. Traumatic events and long-lasting exposure to occupational stressors may be associated with changes in the quantity and quality of sleep in police officers [10,11,12,13]. The reverse effect, that sleep problems increase occupational stress, has also been shown [14].

Studies showed that changes in sleep patterns in police officers may result in safety and health problems [15], including metabolic syndrome [14,16] or its components [17,18]. Reduced sleep duration and sleepiness were also associated with emotional exhaustion [19]. On the contrary, good quality sleep is a resource that renders police officers more resilient to stress [20]. Night work and numerous occupational factors, such as stress and exposure to violence, combined with poor eating habits, reduced physical activity, use of alcohol and stimulants, as well as poor post-employment health checks cause these workers untreated sleep disorders, fatigue, absenteeism, high error rate, and sleepiness [15]. Sleep mediates the relationship between stress and adverse events [14] and therefore sleep hygiene is of the utmost importance in these workers.

The importance of sleep for police officers led to the setting up of a sleep health promotion program in a police unit continuously engaged in law enforcement activities. Our aims were to test whether: (1) there is an association between sleep problems and occupational accidents or near-miss accidents in police officers; (2) a workplace health promotion (WHP) program can improve the quantity and quality of sleep; (3) sleep improvement is associated with reduction of accidents and near-miss. We hypothesized that: (i) workers who participated in the WHP action in the first year would have an improvement of their sleep parameters; (ii) workers who did not participate in the WHP intervention would not improve their quantity and quality of sleep; (iii) sleep improvements brought about by education in the first group of workers would remain stable the following year; (iv) the workers treated in the second year would align their sleep parameters with those of the first year; (v) after the improvement of sleep problems, there would be a reduction of accidents and near-misses in the whole population.

## 2. Materials and Methods

### 2.1. Population and Setting

A study was conducted on an Italian police unit engaged exclusively in riot and crowd control operations. The members of this unit are routinely deployed to control crowds in high-risk sports or political events, natural disasters, or social emergencies, where public safety may be in danger. To take into account the numerous confounding factors that are present in this type of study, we chose a police unit that had been under medical checks for many years and in which the main risk factors were measured.

In 2009, these police workers were invited to take part in a prospective medical surveillance program that demonstrated that occupational exposure to stressors was associated with mental health problems [21], absenteeism [22], and metabolic syndrome [23]. Both quantitative (sleep loss) and qualitative (sleep dissatisfaction, insomnia symptoms) sleep problems played a mediating role in the relationship between stress and the occurrence of metabolic syndrome [14].

The observation of a high frequency of sleep problems in the cohort and the demonstration of a close association between these problems and the appearance of alterations contributing to metabolic syndrome (arterial hypertension, abdominal obesity, increase of triglycerides, reduction of HDL cholesterol, hyperglycemia) led to the creation of a program to promote sleep health, implemented from 2016–2017.

All members of the police unit who had been in continuous service from 2009–2016 were invited to participate in the study. Of these 242 police workers, 218 (90.1%) agreed to take part in the program. Only 2 officers were female and, for homogeneity, they were excluded from subsequent analyzes. Participation was voluntary and free of charge. The health promotion program was included in the current training of workers. The intervention of sleep health improvement was administered in two phases: the first, that began in January 2016, involved half of the workers; while the second, that commenced the following January (2017), involved the remaining half. For ethical and legal reasons, an intervention in the workplace cannot be limited only to some workers and exclude others [9], therefore, the group of workers who did not participate in the promotion program carried out training activities on general occupational hazards. Assignment to the two groups was randomized by drawing lots. After being informed of the voluntary and confidential nature of the study by means of an information leaflet, participants gave written informed consent to take part in this study by signing a form in their personal health file. The questionnaires were anonymous, and participants were identified by a double-blind alphanumeric code. All data were treated in accordance with the Ethical Principles of Psychologists Code of Conduct (American Psychological Association 2002), the ethical standards of the Declaration of Helsinki, and in compliance with the Italian Law on Privacy [24]. The study-plan was approved by the Ministry of the Interior.

### 2.2. Training and Medical Support

A team that included a neurophysiologist and a psychologist, and was led by the physician in charge of the medical surveillance of workers, provided training activities. Evidence-based training modules provided police officers with clinically relevant information to help them modify lifestyle habits that could affect their sleep hygiene. Small groups of 10–20 trainees underwent initial training in two 4-h sessions. The training content was organized into four areas: (1) the medical importance of sleep; (2) the relationship between sleep and wellbeing; (3) sleep apnea and other sleep disorders; (4) sleepiness countermeasures and sleep hygiene. All workers in the two groups received initial training in the months of January and February of each year.

Throughout the training course, trainees learnt about basic sleep needs; the importance of sleep for health and safety; how to identify symptoms of sleep disorders; how to get screened for sleep disorders; and how to combat sleepiness and improve sleep hygiene on a day-to-day basis. The course underlined the importance of taking short naps to prevent sleepiness. The nap, lasting no longer than 30 min, had to be carried out preferably in the afternoon (3–5 p.m.), at least one hour before the work shift [25,26]. Trainers insisted on the importance of sleeping at least 7 h per night. Training was designed to be interactive, and trainers encouraged and guided open discussion about sleep-related questions and concerns, so as to cover all the topics within the two 4-h sessions. Training materials included a power point presentation with videos of case studies and a manual on sleep hygiene that was distributed to the police officers.

A second 4-h training session (booster) was completed in the middle of each year (June–July). On this occasion the workers were asked to check if they had understood the principles indicated in the initial part of the training and, above all, if they had put these into practice. In particular, the trainers stressed the importance of applying the principles of sleep hygiene and taking naps when too tired during extra time and overtime. Workers were invited to reflect on which work factors might interfere with proper sleep hygiene and to seek ways to achieve the best possible adaptation.

All workers were examined by one of the authors of the paper (SG), who is an occupational health specialist, and also a specialist in sleep medicine, at the beginning and end of the observations. During the visit, the doctor limited himself to analyzing the general conditions of the workers and the quantity and quality of sleep. He gave workers who had sleep problems (difficulty falling asleep, frequent sleep interruptions, or early awakenings) or were dissatisfied with the quantity or quality of sleep, additional counseling and reinforced sleep hygiene indications, without intervening with drugs, nor with non-pharmacological treatment, such as cognitive-behavioral treatment for insomnia [27,28]. This medical examination was provided free of charge. None of the police officers were treated with sedative-hypnotic drugs. They cannot take these drugs because they are armed, active 24 h a day and 7 days a week, and their alertness is a top priority.

### 2.3. Design

A pre-post design was used to evaluate the program. At baseline (January/February 2016), both groups of police officers (A and B) underwent medical examination with questionnaires. At the end of the follow-up (November/December 2017), during the final medical examination, a further questionnaire was administered. The biometric health screenings included collection of the following information: date of birth, personal and family history, geographical origin, marital status, presence of offspring, type of dwelling (house or barracks), education level, hierarchical rank, and length of service.

Physical activity was questioned with one question during the medical examination: “How often have you been exercising in the past week? (at least 30 min of gym, bike, football, swimming, running, etc.)”. The answer was graded on a Likert scale, from 0 = never to 5 = 5 or more times per week. The control of the diet was verified through a question during the medical examination: “How often have you tried to limit salt, sugar, or fat in meals in the last week?”. The response was graded from 0 = never, to 4 = in all meals. Alcohol use was investigated by the question: “In the past week, how many glasses have you drunk? (An average glass of wine or a 33 cL bottle of beer or a glass of liqueur)”. The response was graded from 0 = none, I do not drink, to 4 = 14 or more units a week, more than 2 units a day. The consumption of coffee was verified through a question during the medical examination: “How many cups of coffee a day have you taken in the last week?”.

Workers were questioned to ascertain how many in the last month were regularly taking short naps before going on duty, and how many had had an accident at work or a near-miss accident in the previous year. The prevalence (number of cases) of workers who claimed to have the habit of making naps before going on duty in the evening was measured at baseline and after the training. The prevalence of workers reporting injuries was checked using accident records. The occurrence of work injuries and accidents in the previous year was registered from police records both at baseline (for the year 2015) and one year after the conclusion of the program (for the year 2018).

### 2.4. Questionnaire

In compliance with the protocol adopted in the multicenter study SOLARIS (OSAS screening for workers in high risk of injury or accident), which is detailed elsewhere [29], the presence of sleep problems was screened through medical examination and questionnaires on sleep quantity/quality and excessive daytime sleepiness. The answers to the questionnaires were verified during the interview with the doctor. Sleep problems were evaluated with the Sleep Disorder Score questionnaire (SDS-Q) [30], a short validated Italian tool which has been used in previous studies [31,32,33,34]. The questionnaire provided a numerical variable (HS, hours of sleep); one ordinal variable concerning sleep quality, SS (Likert scale graded from 1–4, corresponding to very bad, quite bad, fairly good, very good); and three ordinal variables (DS, difficulty sleeping; SI, sleep interruptions; EA, early awakenings; SS, sleep quality) corresponding to items with a Likert scale graded from 1–4 (never, rarely, sometimes, often), which can be added together to create an insomnia symptom score variable (IS) with continuous values ranging from 3–12. Moreover, for operational purposes, while taking into account the complexities of defining optimal sleep [35], some binary variables were created. The police workers who slept less than 7 h were classified as insufficient sleepers. Similarly, the subjects who attributed grade 3 or 4 to questions DS, SI, and EA were classified as individuals with difficulty falling asleep, sleep interruptions, early awakenings, while those who gave a score of 1 or 2 on the question of sleep quality were classified as being dissatisfied with the quality of sleep.

Sleepiness was evaluated with the Epworth Sleepiness Scale ESS [36] in the Italian validated version [37], a self-administered questionnaire with 8 questions that rated on a 4-point scale (0–3) the chances of dozing off or falling asleep while engaged in eight different activities. An EES score >10 was classified as excessive daytime sleepiness (EDS) [38].

### 2.5. Statistics

The characteristics of subgroups A and B were compared using the chi-square test, the Student *t*-test, and the Mann Whitney–Wilcoxon U test for independent samples. The McNemar test was used to evaluate the existence of differences in dichotomous data (presence/absence) before and after the promotion program. Pre-post comparisons of continuous variables were performed with Student’s *t*-test and Mann Whitney–Wilcoxon U test for paired data.

The effect modification by study groups (A and B) on the time course (i.e., on the magnitude of the differences between the changes observed during the intermediate and the whole follow-up period) of the insomnia symptom score, hours of sleep, sleep satisfaction, and the ESS score was investigated by standard interaction analysis.

Logistic regression was used to assess which sleep characteristics were most associated with injuries and near-misses before and after the program. For each of the outcomes (accident and near-miss), we constructed three series of regression models: the first, univariate, contained each of the sleep-related symptoms (hours of sleep, insomnia symptom score, sleep satisfaction, sleepiness) as the independent variable. The second set of models was adjusted for socio-demographic variables (age, education level, hierarchical rank, geographical origin, type of dwelling, marital status, presence of offspring). The third set of models included all sleep variables.

## 3. Results

### 3.1. Characteristics of the Sample

A count of 218 out of 242 (90.1%) male police officers with a mean age of 42.13 ± 7.33 years (range 29–61), completed the study. All the members of the sample, who had been carefully selected among ordinary police officers and had received specific psychophysical and tactical training, were in good health and did not suffer from diagnosed sleep disorders or other common conditions that required taking drugs that interfere with sleep. None of the officers had obstructive sleep apnea, restless legs syndrome, or other sleep-related disorders.

With reference to physical activity and dietary habits, the results of the baseline medical examination showed the sample was very homogeneous. All workers performed physical activity five or more times a week. Regarding dietary habits, eight workers reported they had controlled diet “in most meals”, the remaining “always, in all meals”. Even with regard to alcohol use, the responses were highly polarized on one extreme of the Likert scale. Forty-two of them (19%) were abstainers, 176 (81%) consumed one to seven units of alcohol per week, that is, one per day, in only one of the meals; no one drank more than seven units per week. A share of the 86% took coffee, but none of them claimed to take three or more cups of coffee a day. Eight workers (five in the A and three in the B group) took antihypertensive drugs for mild hypertension, mainly beta-blockers (two cases) and angiotensin-converting enzyme inhibitors (five cases), which are not reported to interfere with sleep [39].

### 3.2. Comparison of the Sub-Groups

The characteristics of the two groups that participated in the A and B phases of the project did not differ significantly at baseline (Table 1), nor did the prevalence of sleep problems in the two A and B subgroups at the beginning of the observations (Table 1).

After one year, the prevalence of symptoms in subgroup A, which had carried out the health promotion procedure, was significantly lower than that of group B (*p* = 0.003). Compared with baseline data, the workers that had participated in the first phase of intervention reported a significant reduction in the insomnia symptom score and an increase in hours of sleep. Daytime sleepiness and sleep dissatisfaction also declined in subgroup A, although the difference compared with the basic values failed to reach a statistically significant level (Table 2).

In the middle of the program, a significant deterioration in the insomnia symptom score and in sleepiness (Table 2) was observed in subgroup B, which had not undergone treatment. Interaction analysis demonstrated that the study groups (A versus B) significantly modified the time course (i.e., the magnitude of the differences between the changes observed during the intermediate and the whole follow-up period) of the insomnia symptom score (*p* < 0.001) but not that of hours of sleep (*p* = 0.26), sleep satisfaction (*p* = 0.35), and the ESS score (*p* = 0.14).

When group B also carried out the programmed intervention at the end of the second year, the prevalence of sleep problems in the two subgroups returned to comparable levels (Table 1). After intervention, the number of police officers who had a short sleep duration of less than 7 h decreased significantly (*p* < 0.017), while a significant increase was observed (McNemar test *p* < 0.001) in the number of police officers who took naps (*n* = 100, 45.9%) compared with baseline (*n* = 66, 30.3%).

When we consider the entire sample of police officers, the people who reported symptoms indicating poor sleep quality were significantly reduced at the end of follow-up (Table 3). Officers reporting satisfaction with sleep had increased (*p* = 0.005), and those who reported daytime sleepiness had decreased significantly (*p* = 0.031). In the same period, there was a significant reduction in the frequency of injuries and near-misses compared with the data reported at baseline. Compared with baseline, a very significant difference was found in 2017, particularly in the reporting of near-miss accidents in the 3-month period before medical examination (McNemar test *p* = 0.006). The number of police officers having at least one working accident in 2018 was significantly lower than in 2015 (McNemar test *p* = 0.026).

Logistic regression analysis was used to study the association between sleep variables and injuries in the whole group. In the univariate models, all the variables investigated were very significantly associated with the presence of near-misses at baseline; the strength of the association remained unchanged with the introduction of demographic and social variables. At baseline, in the multivariate model, in which all the variables were entered as predictors, only sleep satisfaction retained a significant protective effect on the near-misses, but the variable most significantly associated with near-misses was excessive daytime sleepiness (OR = 1.382; CI95% 1.182–1.615).

In the cross-sectional analysis of the data recorded at follow-up, the association between hours of sleep and near-misses barely reached statistical significance, while all other variables related to sleep were very significantly associated with near-misses in univariate logistic models. In the final multivariate model, sleep satisfaction and insomnia symptoms were the best predictors of near-miss events (Table 4).

The same type of relationship described above for near-misses was found at baseline for the association between sleep variables and occupational accidents. All sleep-related variables were associated with injuries, but the strongest association in the multivariate model at baseline was between sleepiness and accidents (OR 1.220; CI95% 1.044–1.426).

In the analysis of data at follow-up, all sleep variables were significantly associated with injuries, but in the multivariate model III, only insomnia symptoms were found to have a significant predictive relationship with work accidents (OR 13.358; CI95% 2.353–75.818). The latter prediction model has a value for the coefficient of determination (Nagelkerke’s R squared = 0.818) close to unity; this indicates that most of the variance of accidents at work that would have occurred in the year following the implementation of the program could be explained by an equation that includes socio-demographic and sleep variables.

Prior to intervention, excessive daytime sleepiness was the main predictor of accidents at work and near-miss accidents. After training, the number of workers who had the habit of taking naps increased, while those with sleep problems decreased. Daytime sleepiness also declined and was no longer the main factor associated with injuries and near-misses, while the association between sleep quality and these events was highly significant.

In the logistic regression model, correction for the confounding variables (age, geographical origin, marital status, presence of offspring, type of dwelling, education level, hierarchical rank, length of service) did not substantially change the associations, although, as was expected, the larger number of variables and the collinearity between sleep variables extended the confidence intervals, both for near-miss accidents (Table 4) and occupational injuries (Table 5).

## 4. Discussion

Our study indicates that a WHP program that combined training and medical non-pharmaceutical interventions resulted in a significant improvement in sleep health in a group of police officers engaged in maintaining law and order. The accidents and near-miss accidents that occurred before the WHP program correlated with sleep problems.

After intervention, a reduction was also observed in the frequency of accidents at work and near-miss accidents. The causal association between the two phenomena cannot be proven but is highly probable, given the association demonstrated between sleep loss and injuries in previous studies [40,41].

In agreement with our hypotheses (i), (ii), and (iv), improvements in the quantity and quality of sleep were evident in subgroup A in the first year, while sleep parameters in the second group, that had not commenced the WHP program, were stable or had worsened. After participating in the WHP program, the benefits were also evident in subgroup B, confirming hypothesis (v). The improvements obtained in subgroup A in the first year remained stable also at the end of the second year, according to hypothesis (iii).

Our findings must be interpreted with caution on account of the limited number of observations and the specific professional profile of the sample. The characteristics of this selected population do not mirror that of all police forces. Indeed, the members of the cohort did regular physical exercise, including mandatory physical-technical-tactical activities (8 h per month) in addition to gym exercises in the barracks, jogging, running, cycling, CrossFit, and other physical activities for 9–12 h a week. They ate in a canteen where the same menus, based on a correct nutritional intake, were available for all the staff. No use of alcohol is permitted during work. As a result, all were moderate drinkers or non-drinkers. Work shifts, which often required overtime, were rotated, but there were no night shifts. Workers executed a “ready-to-work” schedule of 6 h, awaiting a call for public order and/or organized work services; extra- and overtime was limited to a maximum of 8 h per day (max 36 h a week), and could also be nocturnal. The workers of this selected police unit had good health conditions, better than those found in other experiences of these workers [42]. None of them suffered from obstructive sleep apnea syndrome, which is quite common among police officers [43], and all had good physical activity and a regulated diet. They did not use sleep- or wake-promoting drugs, which is a fairly frequent condition in police forces [44], Moreover, although they may have irregular shifts, they do not work night shifts, which are associated with poor sleep quality [45], altered cognitive performance [46], and disturbed feeding patterns [47] in police officers. Our observations, therefore, cannot be extended to all the police forces, but only to these special units devoted to the control of public order.

However, our findings could be useful for subsequent studies on the subject. Moreover, this experience suggests that a workplace health sleep promotion program, based on a relatively modest use of medical and educational resources, can achieve significant improvements in workers’ health and safety. The use of logistic regression to study the association between the quantity and quality of sleep and near-misses or accidents at work provided indications that merit further verification on larger populations. At the beginning of our observations, when the workers had very low levels of quantity and quality of sleep, the near-miss accidents and the injuries that occurred in the previous year were significantly associated with excessive daytime sleepiness. After the training course, the police officers’ sleep hygiene improved significantly, as did their habit of taking naps. Better sleep hygiene led to a lower frequency of sleep deprivation and sleepiness, and events were significantly associated mainly with the quality of sleep and symptoms of insomnia, i.e., difficulty in falling asleep, frequent interruptions in sleep, and awakenings. The insomnia symptoms that were still present at the end of the program probably require medical treatment and their association with injuries and near-misses calls for further intervention.

The results of this study are consistent with the literature. All markers of sleep disorders are associated with an increased risk of injuries at work [48]. Excessive daytime sleepiness is an important determinant of driving accidents [49], regardless of the simultaneous presence of sleep disorders or sleep deprivation [50]. According to data from a recent meta-analysis [51], the risk of accidents at work in individuals who suffer from sleep disorders is at least double compared with healthy workers.

Our study has many limitations. First of all, the limited size of the sample, the lack of gender, racial, and ethnic heterogeneity, and the specific characteristics of the police activity carried out by this special unit prevented the results from being applicable for the police profession as a whole. Nevertheless, the results obtained in this police unit, specifically engaged in the control of law and order, can be extended to similar units present in Italy and in other countries. Secondly, as no external control group was used, it was not possible to ascertain whether sleep benefits and the accident rate reduction were a consequence of participation in the study or some other confounding factor that was not taken into consideration in our design. However, since the improvement in sleep parameters coincided with the intervention, and sleep disorders and especially symptoms of insomnia and sleepiness tended to increase in group B, initially left without intervention, it is reasonable to attribute the observed effect to the training and medical support offered. Thirdly, the statistical model used to study the relationship between the various measures of sleep problems and accidents and near-miss suffers a high degree of collinearity between the variables and must therefore be considered indicative. Finally, our measurements were based only on self-reported survey data, where recall bias or observer effect are potential limitations. However, it is important to note that subjective ratings of sleep satisfaction are generally considered valid in the literature and provide a reliable measure of the effectiveness of intervention. Our conclusion is that an effective health promotion action can be carried out in the police force without excessive expenditure of material and human resources.

The importance of occupational problems related to poor sleep quality and daytime sleepiness have led the American Association of Occupational and Environmental Medicine ACOEM [52] and the Italian Society of Occupational Health SIML [53] to suggest that companies introduce a specific sleep risk management program as part of the more general occupational risk management system. This program would include measures designed to combat sleep disorders, and others aimed at improving the state of work alertness. Proper management of staff and work shifts, a thorough and continuous training procedure for workers, managers and supervisors, and lastly the implementation of a reporting system are also recommended. In addition, the program should adopt environmental measures to reduce the risk of causing adverse effects due to sleepiness. Individual protection against sleep disorders should also be improved. Workers, colleagues, and supervisors should look out for early signs of excessive sleepiness and introduce timely countermeasures. Finally, the program should be constantly monitored and undergo periodic review. Screening of sleep disorders can be usefully included in the activities of the physician in charge of the medical surveillance of workers (the so-called “competent doctor”) without a significant loss of time, and with increased worker satisfaction.

The Guidelines for the Health Surveillance of Police Forces [54], recently implemented in Italy, underline the importance of sleep hygiene.

The aforementioned intervention is too recent to enable us to claim that the improvements observed are permanent. The messages transmitted during the training course need to be repeated during the medical examinations police officers regularly undergo. However, this study supports those who believe that occupational health surveillance should not be limited to the prevention of risks arising directly as a result of performing work tasks, but should be extended to other aspects of life, including the prominent role of sleep.

## 5. Conclusions

This study showed that a sleep health promotion intervention can improve the occupational health of police officers. Reductions in daytime sleepiness and improvement of the quantity and quality of sleep were associated with a reduction in workplace injuries. The promising results and the modest cost of an intervention that has been included in the usual worker training program should induce others to repeat the experience and verify the results. The prevention of sleep problems is of primary importance for the health and safety of workers. Occupational health and safety services should develop programs to promote sleep health in the workplace.

## Figures and Tables

**Table 1:** Difference between A and B subgroups of workers at beginning, after the first wave of intervention (first year), and at follow-up.

Baseline	Group A (*n* = 114)	Group B (*n* = 104)	p *
Age	41.6 ± 6.95	42.7 ± 7.72	0.291
Rank, graduated (N)	65	54	0.451
Education, superior (N)	82	77	0.726
Birthplace, Northern Italy (N)	62	50	0.352
Married (N)	42	47	0.210
Barracks (N)	53	54	0.423
Children(N)	41	49	0.095
Hours of sleep	6.59 ± 1.14	6.64 ± 1.09	0.709
Insomnia symptom score	6.05 ± 1.85	5.96 ± 1.81	0.744
Sleep satisfaction	3.07 ± 0.90	3.24 ± 0.99	0.081
ESS score	6.10 ± 3.89	6.13 ± 3.70	0.844
**Intermediate-1st year**			
Hours of sleep	6.84 ± 0.99	6.58 ± 1.09	0.062
Insomnia symptom score	5.41 ± 1.49	6.13 ± 1.86	0.005
Sleep satisfaction	3.21 ± 0.81	3.16 ± 1.03	0.803.
ESS score	5.74 ± 3.25	6.25 ± 3.71	0.321
**Follow-up-2nd year**			
Hours of sleep	6.82 ± 0.93	6.72 ± 0.93	0.414
Insomnia symptom score	5.41 ± 1.55	5.14 ± 1.33	0.290
Sleep satisfaction	3.20 ± 0.80	3.36 ± 0.80	0.119
ESS score	5.75 ± 3.19	5.64 ± 3.09	0.938

(*) Student’s *t*-test for independent samples (hours of sleep, age), Mann Whitney–Wilcoxon U test for independent samples (non-parametric variables: insomnia symptom score, sleep satisfaction, ESS score), Pearson’s chi square test for categorical data.

**Table 2:** Changes of sleep variables during the intervention.

Whole Group	Baseline	Intermediate	p *	Follow-Up	p *
Hours of sleep	6.61 ± 1.12	6.72 ± 1.05	0.021	6.78 ± 0.93	0.001
Insomnia symptom score	6.01 ± 1.83	5.75 ± 1.71	0.001	5.28 ± 1.45	0.000
Sleep satisfaction	3.15 ± 0.95	3.19 ± 0.92	0.322	3.28 ± 0.80	0.010
ESS score	6.11 ± 3.79	5.98 ± 3.48	0.635	5.70 ± 3.14	0.030
**Group A**					
Hours of sleep	6.59 ± 1.14	6.84 ± 0.99	0.001	6.82 ± 0.93	0.001
Insomnia symptom score	6.05 ± 1.85	5.41 ± 1.49	0.000	5.41 ± 1.55	0.000
Sleep satisfaction	3.07 ± 0.90	3.21 ± 0.81	0.053	3.20 ± 0.80	0.056
ESS score	6.10 ± 3.89	5.74 ± 3.25	0.302	5.75 ± 3.19	0.270
**Group B**					
Hours of sleep	6.64 ± 1.09	6.58 ± 1.09	0.090	6.72 ± 0.93	0.278
Insomnia symptom score	5.96 ± 1.81	6.13 ± 1.86	0.010	5.14 ± 1.33	0.000
Sleep satisfaction	3.24 ± 0.99	3.16 ± 1.03	0.102	3.36 ± 0.80	0.085
ESS score	6.13 ± 3.70	6.25 ± 3.71	0.033	5.64 ± 3.09	0.046

(*) Student’s *t*-test for paired data (hours of sleep), Mann Whitney–Wilcoxon U test (non-parametric variables: insomnia symptom score, sleep satisfaction, ESS score).

**Table 3:** Comparison of the prevalence of sleep problems, habit of taking naps, accidents, and near-misses at baseline and at the end of the follow-up (whole group).

Disorder	Baseline (*n*, %)	Follow-Up (*n*, %)	Mc Nemar Test *p*
Difficulty of falling asleep	49 (22.5)	34 (15.6)	0.012
Sleep interruptions	72 (33.0)	57 (26.1)	0.018
Early awakenings	60 (27.5)	46 (21.1)	0.011
Unsatisfactory sleep	63 (28.9)	44 (20.2)	0.005
Short sleep duration (<7 h)	102 (46.8)	85 (39.0)	0.017
Daytime sleepiness	27 (12.4)	15 (6.9)	0.031
Habit of making naps	66 (30.3)	100 (45.9)	0.000
Workers reporting at least 1 near-miss accident	37 (17.0)	18 (8.3)	0.006
Workers having at least 1 working accident	26 (11.9)	13 (6.0)	0.026

**Table 4:** Logistic regression analysis. Univariate association of sleep variables with near-miss accidents in police officers, at baseline and after the intervention (whole group).

	Near-Miss Accidents		
Baseline	Model I OR (CI 95%)	Model II OR (CI 95%)	Model III OR (CI 95%)
Hours of sleep	0.503 (0.354–0.714) ***	0.500 (0.349–0.717) ***	1.086 (0.654–1.806)
Insomnia symptom score	1.429 (1.175–1.737) ***	1.398 (1.145–1.707) ***	0.974 (0.743–1.277)
Sleep satisfaction	0.355 (0.236–0.534) ***	0.357 (0.233–0.546) ***	0.486 (0.267–0.882) **
ESS score	1.451 (1.272–1.656) ***	1.466 (1.273–1.689) ***	1.382 (1.182–1.615) ***
		Nagelkerke’s R squared	0.404
**Follow-up**			
Hours of sleep	0.558 (0.321–0.967) *	0.542 (0.307–0.956) *	1.070 (0.573–1.999)
Insomnia symptom score	2.038 (1.469–2.827) ***	2.049 (1.448–2.899) ***	1.652 (1.115–2.450) *
Sleep satisfaction	0.145 (0.063–0.332) ***	0.127 (0.052–0.307) ***	0.161 (0.063–0.412) ***
ESS score	1.266 (1.071–1.496) ***	1.264 (1.063–1.503) ***	1.081 (0.856–1.365)
		Nagelkerke’s R squared	0.436

(***) *p* < 0.001; (**) *p* < 0.01; (*) *p* < 0.05; Model I: univariate association; Model II: adjusted for age, education level, hierarchical rank, geographical origin, type of dwelling (house or barracks), marital status, presence of offspring; Model III: additionally, adjusted for all sleep variables.

**Table 5:** Logistic regression analysis. Univariate association of sleep variables with work accidents in police officers, in 2015 and 2018.

Baseline	Work Accidents		
	Model I OR (CI 95%)	Model II OR (CI 95%)	Model III OR (CI 95%)
Hours of sleep	0.521 (0.352–0.770) ***	0.524 (0.353–0.778) ***	1.109 (0.637–1.929)
Insomnia symptom score	1.466 (1.174–1.832) ***	1.433 (1.142–1.798) ***	1.102 (0.825–1.473)
Sleep satisfaction	0.393 (0.250–0.617) ***	0.379 (0.237–0.605) ***	0.505 (0.266–0.959) *
ESS score	1.344 (1.180–1.531) ***	1.328 (1.160–1.520) ***	1.220 (1.044–1.426) ***
		Nagelkerke’s R squared	0.293
**Follow-up**			
Hours of sleep	0.440 (0.227–0.853) *	0.330 (0.142–0.767) **	0.469 (0.080–2.752)
Insomnia symptom score	4.068 (2.352–7.035) ***	11.75 (2.875–48.021) ***	13.358 (2.353–75.818) ***
Sleep satisfaction	0.365 (0.183–0.731) ***	0.315 (0.131–0.757) **	1.526 (0.193–12.085)
ESS score	1.488 (1.193–1.857) ***	1.518 (1.149–2.005) ***	1.299 (0.716–2.360)
		Nagelkerke’s R squared	0.818

(***) *p* < 0.001; (**) *p* < 0.01; (*) *p* < 0.05; Model I: univariate, unadjusted; Model II: adjusted for age, education level, hierarchical rank, geographical origin, type of dwelling (house or barracks), marital status, presence of offspring; Model III: additionally, adjusted for other sleep variables.

## Data Availability

The data that support the findings of this study are openly available in Zenodo at https://zenodo.org/record/3744746#.X0p24-9xeUk [doi md5:3402e5847676d55bb16c77deda638f42].
